# Real-world efficacy and safety of eribulin in advanced and pretreated HER2-negative breast cancer in a Spanish comprehensive cancer center

**DOI:** 10.1186/s40360-019-0367-x

**Published:** 2019-11-21

**Authors:** Milana Bergamino Sirvén, Adela Fernández-Ortega, Agostina Stradella, Idoia Morilla, Catalina Falo, Silvia Vázquez, Roser Castany, Rafael Villanueva, Sabela Recalde, Valentí Navarro Pérez, Miguel Gil-Gil, Sonia Pernas

**Affiliations:** 1 Department of Medical Oncology-Breast Cancer Unit, Insitut Catala d’Oncologia (ICO)-H.U.Bellvitge- IDIBELL, Avinguda Gran Via 199-203, 08908-L’Hospitalet de Llobregat, Barcelona, Spain; 2 Research Unit, Institut Català d’Oncologia-(ICO) L’Hospitalet, Barcelona, Spain

**Keywords:** Eribulin, Breast cancer, Real-world data, Brain metastases, Obesity, Progression pattern, Elderly patients

## Abstract

**Background:**

Eribulin improves survival in pre-treated HER2-negative advanced breast cancer (ABC). However, limited data exist on co-morbidities and central nervous system (CNS) efficacy. The purpose of this study was to review eribulin’s efficacy and safety in everyday clinical practice with special focus on age, body mass index (BMI) and central nervous system (CNS) activity.

**Methods:**

An observational study was conducted in a series of HER2-negative ABC patients treated from January’14-December’17 outside a clinical trial. Objective Response Rate (ORR), Progression Free Survival (PFS), Overall Survival (OS), and association of clinical and pathological variables with outcome were evaluated.

**Results:**

Ninety-five women were treated with at least one cycle of eribulin. Median age was 57 (33–83), and 18% were obese. Median number of prior chemotherapies for ABC was 3 (2–5) and 76% of patients had visceral metastases, including 21% with CNS involvement. Most tumors were estrogen receptor-positive (79%). ORR and stable disease (SD) at 6 months were 26.2 and 37.5%, respectively. Remarkably, relevant CNS efficacy was observed with eribulin: 20% of patients obtained partial response and 25% SD. Treatment was generally well tolerated and manageable, with 29% grade 3 and 10.9% grade 4 toxicities. Median PFS and OS were 4.1 months (CI95% 3.2–4.9) and 11.1 months (CI95% 9.5–14.7), respectively. Triple-negative disease, > 2organs involved and being younger than 70 years old were independent prognosis factors for worse OS in multivariate analysis. Most patients (75%) progressed in pre-existing metastases sites.

**Conclusion:**

In everyday clinical practice, eribulin’s efficacy seems similar to pivotal trials. CNS-efficacy was observed. TNBC, > 2 organs involved and being younger than 70 years old were independent prognosis factors for worse OS. Remarkably, less incidence of grade 4-toxicity compared to previous studies was found.

## Background

Eribulin mesylate is a synthetic analogue of the marine halicondrin B, a microtubule-targeting agent approved for the treatment of metastatic breast cancer. Some preclinical data showed that eribulin can reverse the epithelial–mesenchymal transition (EMT), reducing cancer cell migration and invasion [1, 2]. Additionally, eribulin has an antiangiogenic effect and it can cause tumor vascular remodeling, leading to an increased perfusion into the tumor and metastases [3–5].

Two phase III randomised studies (EMBRACE and 301 trials) and a pooled analysis of these two studies showed that eribulin improved overall survival (OS) in patients with pretreated advanced breast cancer (ABC) [6–8]. The benefits of eribulin were achieved with a manageable toxicity profile in both studies. The beneficial effects of eribulin compared with the control were mainly observed in patients with triple-negative breast cancer (TNBC) or HER2-negative disease, in patients with > 2 organs involved and in patients with visceral disease [8]. Moreover, a reduced incidence of new location metastases when patients are treated with eribulin has been reported in some retrospective studies, probably due to its biological effect [9–11]. In contrast, few data exist about prognostic and predictive factors of response with eribulin in everyday clinical practice [12, 13] . Efficacy in elderly patients, obese patients, or in patients with central nervous system (CNS) disease has not been well reported either [14–16].

Eribulin was approved in Spain in December 2013 for patients with HER2-negative recurrent or metastasic breast cancer previously treated with taxanes, anthracyclines and capecitabine unless patients were not suitable for those treatments [17–19]. The purpose of our study was to review eribulin’s activity, in terms of progression-free survival (PFS) and OS, and tolerability in patients with HER2-negative ABC treated at ICO-Hospitalet (Barcelona, Spain) outside of a clinical trial since its approval.

## Methods

### Study population

An observational study was conducted at ICO-Hospitalet to review patients with HER2-negative ABC consecutively treated with single-agent eribulin from January 2014 to December 2017 in an off-trial setting. All patients included may have received at least one cycle of eribulin 1.23 mg/m2 day 1 and 8 every 21 days. The treatment was continued until disease progression, unacceptable toxicity, or patient withdrawal. Age at diagnosis, body mass index (BMI), relevant comorbidities, Eastern Cooperative Oncology Group (ECOG) Performance Status, tumor subtype, visceral involvement, pre and post-eribulin chemotherapy regimens, response rate, clinical benefit, CNS efficacy, toxicities and pattern of progression were obtained from medical records. Toxicity was reported following the Common Terminology Criteria for Adverse Events (CTCAE 3.0).

Regarding comorbidity, we did not use the Charlson Comorbidity Index due to the strong influence of the presence of metastases (M1) on this scale, but presence of relevant comorbidity used in that scale was registered and graded, and we considered it relevant if patients had one or more comorbidities with grade 2 or higher. In this study the comorbidity that was considered and graded was: diabetes mellitus, hypertension, dyslipidemia, myocardial disease such as myocardial infarction and congestive heart failure, chronic pulmonary disease, liver and renal disease.

### Statistical analysis

The characteristics of patients were described and the Kaplan–Meier method was used to calculate the PFS and OS as the time-to-event for each endpoint from the start date of treatment with eribulin to the occurrence date of the event, progression or death, respectively. An evaluation of the response was performed by the response evaluation criteria in solid tumors (RECIST 1.1.). Objective response rate (ORR) included complete response (CR) and partial response (PR) at the first evaluation (generally performed every 9–12 weeks or if clinically indicated). Clinical benefit rate included CR, PR or stable disease (SD) of at least 6 months’ duration.

Statistical differences were estimated using the log-rank test. The univariate and multivariate analyses were conducted using the Cox proportional hazard regression model for PFS and OS to assess the prognostic value of patients, tumors and treatment characteristics. A two-tailed *p-*value of < 0.05 was considered significant. In the multivariate analysis, we included the variables that achieved a *p*-value < 0.1 in the univariate analysis. All analyses were carried out using the SPSS 22.0 statistical software (SPSS Inc., Chicago, IL, USA).

## Results

### Patient characteristics

Ninety-five women with HER2-negative ABC were treated with at least one cycle of eribulin during this period. Baseline patient and tumor characteristics are listed in Table 1. Overall, median age was 57 years (range 33–83), ECOG Performance Status was ≤1 in 88% of patients and only 12.6% of patients had relevant comorbidities. Median number of organs involved was 3 (range 1–5). Of those patients with CNS involvement (*n* = 20), seven (33%) had been previously treated with whole brain irradiation. In patients pretreated with taxanes, 46% progressed during the first 3 months and they were considered taxane-refractory, based on previous studies [7].

**Table 1:** Baseline characteristics of the patients included in the study (*n* = 95)

Variable	*N*	%
Age		
< 70 years old	79	(83%)
≥ 70 years old	16	(17%)
Comorbidities		
Hypertension	13	(13,7%)
Dyslipidemia	9	(9.5%)
Diabetes Mellitus	3	(3.2%)
Cardiorespiratory disease	9	(9.5%)
Liver and renal disease	5	(5.3%)
Relevant comorbidity (**≥** grade 2)	12	(12.6%)
BMI		
BMI < 25	43	(45%)
BMI 25–30	35	(37%)
BMI > 30	17	(18%)
Histology		
Infiltrating ductal carcinoma	84	(88%)
Infiltrating lobular carcinoma	11	(12%)
Tumor subtype		
ER and/or PgR positive	75	(79%)
Triple Negative	16	(17%)
HER2-positive	4	(4%)
Previous chemotherapies		
Taxane	93	(98%)
Capecitabine	93	(98%)
Vinorelbine	33	(35%)
Location of M1		
Bone	73	(77%)
Lymph nodes	67	(71%)
Liver	62	(65%)
Lung	29	(31%)
Pleura	23	(24%)
Central nervous system	20	(21%)
Skin	19	(20%)
Visceral metastases		
Yes	72	(76%)
No	23	(24%)
Number of organs involved		
≤ 2 organs	35	(37%)
> 2 organs	60	(63%)

Abbreviations**:** *BMI* body mass index, *ER* estrogen receptor, *PgR* progesterone receptor

### Efficacy of eribulin according to patient and tumor characteristics

Median number of cycles of eribulin received was 7 (1–55). Dose interruptions, delays and reductions were 14, 26 and 32%, respectively. ORR was 26.2% (all of them PR) and 37.5% of patients had SD, with a clinical benefit observed in 63.7% of patients. Regarding CNS efficacy, 4 patients (20%) presented a PR, 5 patients (25%) achieved SD and 2 patients were not evaluated. Remarkably, out of the nine patients with CNS metastases who progressed during treatment with eribulin, six (66%) had meningeal carcinomatosis. In the subgroup of patients without previously known CNS disease, only two patients progressed to meningeal carcinomatosis during treatment with eribulin. Disease control seemed independent to previous CNS local treatment. From the seven patients who had been previously treated with whole brain irradiation, two achieved PR, three SD and two progressed during treatment with eribulin.

A different pattern of ORR was observed in patients based on age (< 70 years vs ≥70 years). Clinical benefit was 62% in the younger group of patients and 51.3% in the older patients, non-statistically significant (*p* = 0.299), with 25% vs 31.3% achieving PR and 37% vs 20% achieving SD, respectively.

**Fig. 1:**
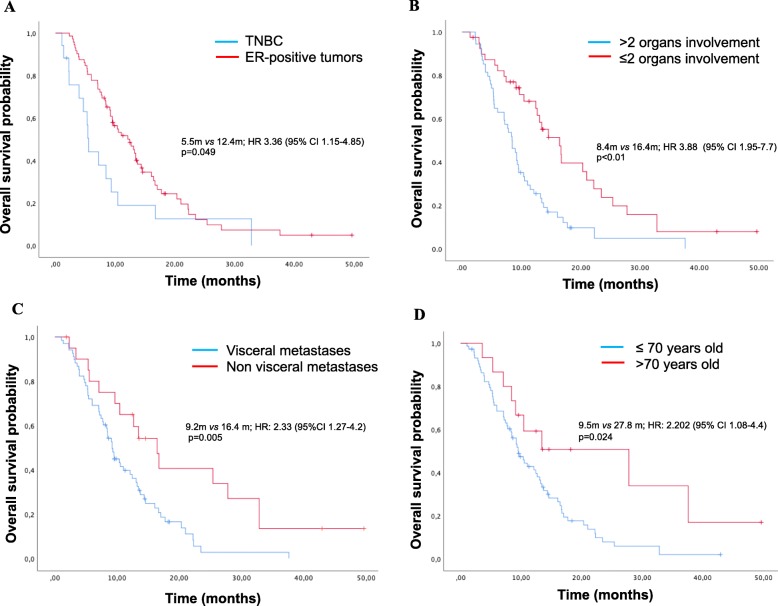
Kaplan Meier curves for overall survival. **a** KM curves for TNBC compared to HR-positive tumors. Median OS was significantly shorter in patients with TNBC compared to ER-positive tumors. **b** KM for OS for burden of disease (< 2 organs involved compared to > 2 organs involved). mOS was significantly shorter in patients with more than 2 organs involved compared to those with 2 or less M1. **c** KM for OS for patients with visceral involvement compared to non-visceral involvement. mOS was significantly shorter in patients with visceral involvement compared to those without visceral M1. **d** KM curves for OS for patients younger than 70 years of age compared to older than 70 years of age. mOS was significantly shorter in patients younger than 70 years of age compared to those older. Abbreviations: KM: Kaplan Meier; OS: overall survival; TNBC: triple-negative breast cancer; m: months; ER: estrogen receptor; mOS: median overall survival; CI: confidence interval; M1: metastases; HR: hazard ratio; m: months

At a median follow up of 13 months, 16 patients (17%) were still alive. Median PFS was 4.1 months (95% CI 3.2–4.9) and was significantly shorter in patients with more than 2 organs involved (3.3 vs 5.9 months; HR 2; 95% CI 1.22–3.26, *p* = 0.01); in patients with TNBC compared to those with HR-positive tumors (3.3 vs 4.5 months; HR 4.16; 95% CI 3.2–4.9, *p* = 0.041); in patients with visceral involvement (3.8 vs 6.2 months; HR 5.35; 95% CI 3.2–4.9, *p* = 0.021); and in patients < 70 years of age (3.8 vs 6.3 months; HR 1.82; 95% CI 1.02–3.25, *p* = 0.011). Median OS was 11.1 months (95% CI 9.5–14.67) and, similar to what was observed with PFS, OS was significantly shorter in patients with TNBC compared to those with HR-positive tumors (5.5 vs 12.4 months; HR 3.36; 95% CI 1.15–4.85, *p* = 0.049) **(**Fig. 1a**)**; in patients with more than 2 organs involved (8.4 vs 16.4 months; HR 3.88; 95% CI 1.95–7.7, *p* < 0.01) **(**Fig. 1b**)**; in patients with visceral involvement (9.2 vs 16.4 months; HR 2.33; 95%CI 1.27–4.2, *p* = 0.005) **(**Fig. 1c**)**; and in patients < 70 years of age (9.5 vs 27.8 months; HR 2.202; 95% CI 1.08–4.4, *p* = 0.024) **(**Fig. 1d**)**. Median OS was worse for taxane-refractory patients (7 vs 12.4 months; HR 1.5; 95% CI 0.406–1.041, *p* = 0.070) and better for patients who experienced grade 3 or 4 toxicities (13 vs 9.5 months; HR 1.5; 95% CI 0.916–2.6, *p* = 0.066), but these differences were not statistically significant. No differences in PFS or OS were observed based on comorbidity or weight.

In the multivariate analysis, only TNBC (HR 2.33; 95% CI 1.25–448, *p* = 0.011), > 2 organs involved (HR 2.27; 95% CI 1.18–4.27, *p* = 0.009) and < 70 years of age (HR 2.35; 95% CI 1.07–5.18, *p* = 0.033) were independent prognostic factors for worse OS (Table 2).

**Table 2:** Multivariate analysis for OS and basal patient and disease characteristics

	HR	95% CI for HR		*P* value
		Inferior	Superior	
Triple negative	2.334	1.215	4.481	0.011
> 2 organs involvement	2.272	1.19	4.274	0.013
Age (< 70 years old)	2.354	1.070	5.180	0.033
Visceral metastases	2.35	0.236	1.259	0.155
Taxane-refractary	0.977	0.566	1.688	0.934
Grade 3–4 toxicities	1.723	0.966	3.072	0.065

Abbreviations: *OS* overall survival, *HR* hazard ratio, *CI* confidence interval

### Toxicity

Toxicities are listed in Table 3. Twenty-nine percent of patients suffered grade 3 adverse events (AE) and 10.9% a grade 4 AE (all neutropenia). The most frequent grade 3 and 4 AEs were asthenia, neurotoxicity and neutropenia and were mainly manageable. All grade 3 and 4 toxicities were independent of a patient’s baseline characteristics (age, obesity, tumor subtype, visceral involvement or number of previous chemotherapy regimens). One patient with grade 4 febrile neutropenia with a skin infection died due to septic shock.

**Table 3:** Most common adverse events following the Common Terminology Criteria for Adverse Events (CTCAE 3.0)

Grades	Neurotoxicity	Alopecia	Asthenia	Thrombocytopenia	Anaemia	Neutropenia	Oral Mucositis	Hepatotoxicity
1	17 (18.5%)	13 (14.1%)	36 (39.1%)	5 (5.4%)	17 (18.5%)	4 (4.3%)	10 (10.9%)	15 (16.3%)
2	18 (19.6%)	11 (12%)	37 (40.2%)	2 (2.2%)	6 (6.5%)	6 (6.5%)	3 (3.3%)	2 (2.2%)
3	7 (7.6%)	–	9 (9.8%)	1 (1.1%)	0	9 (9.8%)	1 (1.1%)	2 (2.2%)
4	0	–	0	0	0	10 (10.9%) ^a^	0	0

^a^ Nine out of ten had febrile neutropenia

### Pattern of progression

Most patients (*n* = 72; 75%) progressed in pre-existing sites of metastases and only 19 (20%) patients experienced disease progression in new locations. Four patients out of those 19 (21%) developed meningeal carcinomatosis. The remaining 5% of patients had clinical progression without radiological evidence of disease progression. The main cause of treatment release was progression (87%) followed by toxicity (13%). Median number of lines of chemotherapy received after eribulin was 1 (0–5).

## Discussion

In the present analysis of the efficacy of eribulin in an off-trial setting, PFS and OS in patients with heavily pretreated HER2-negative advanced breast cancer were similar to those observed in the phase III EMBRACE trial (4.1 vs 3.7 months and 11.1 vs 13.1 months, respectively) [6–8]. Patient characteristics in our study more closely resemble the patient characteristics in the EMBRACE study population than the 301 study population, as most patients had been previously treated with anthracyclines, taxanes and capecitabine [6]. In contrast, the response rate and clinical benefit in our study were higher than in the EMBRACE trial and more similar to the 301 trial results [7]. There was an increased risk of mortality in patients with TNBC compared to HR-positive disease, given the aggressiveness of this tumor subtype. In the pooled analyses, longer median OS was observed in patients with more than two organs involved and in those with visceral metastases [8]; however, in our study, more benefit and longer OS were seen in patients with lower burden of disease and fewer than two organs involved. The more favourable results obtained in these subgroups of patients may reflect their independent better prognosis [8, 12, 20].

In previous reports, patient age had not been described as an independent factor for PFS or OS. A pooled analysis of 827 patients treated in either the EMBRACE trial or the preceding phase II studies reported outcomes according to age. A total of 10% of patients who were included in these studies were aged ≥70 years. No significant differences were observed in ORR, PFS, or OS by age. Toxicity was similar across all age groups, although the incidence of grade 3 and 4 fatigue and peripheral neuropathy was highest in patients aged ≥70 years [14, 21]. Elderly patients with good ECOG-Performance Status treated with eribulin have also shown similar OS, PFS, response rate, clinical benefit and tolerability in smaller studies when compared to younger patients [21, 22]. In our cohort of patients, we observed significant differences in OS according to age, with significantly better OS and PFS in patients ≥70 years of age compared to those younger. Similar ORR but with a distinct pattern regarding PR or SD and tolerability were observed in patients in both age groups.

The role of BMI and comorbidities in the outcomes of patients treated with eribulin is still a matter of debate. In some historical series, patients who are overweight and obese have shown worse outcomes compared to the normal weight group [20, 23, 24]. In an Italian observational multicentric study, there was a significantly better PFS in the lowest category of BMI, but no differences in OS were observed [25]. In our cohort, no differences in OS were found amongst patients with normal weight compared to overweight and obese patients or those with and without comorbidities.

In our study, 21% of patients had CNS involvement, which is often an exclusion criterion in most clinical trials, unless patients are previously treated with local therapy. Of those patients, 25% achieved SD and 20% achieved PR, which supports the efficacy of eribulin in patients with CNS involvement. The efficacy in patients with CNS disease was not related to previous treatment with whole brain radiotherapy. The rationale for potential positive effect of eribulin on brain metastases is not clear. Preclinical evidence suggests that the agent does not cross the blood–brain barrier (BBB) to a significant extent [15, 26]. Radiation therapy can compromise the BBB, which may help drugs enter into the brain [27]. However, this BBB drug crossing can be also explained by the altered BBB due to the presence of brain metastases alone. In the 301 study, a lower proportion of patients in the eribulin arm developed CNS metastases compared to the capecitabine arm (2.4% vs 4.6%, respectively) [7]. Moreover, in the three patients with known stable brain metastases included in that trial and treated with eribulin, a reduction in the size of brain lesions was also observed. Some case reports have also shown efficacy with eribulin at the CNS level, both in patients previously treated with radiotherapy and not [28, 29].

The pattern of disease progression in patients treated with eribulin is distinctive. Most patients in our series progressed in pre-existing M1 sites (75%). This fact may reflect the inhibitory effect of eribulin in the EMT, reducing cancer cell migration and invasiveness; however, we did not find any information about the pattern of progression in patients who received other oncospecific treatments [1–3]. It has been previously reported that patients with ABC who develop tumour progression with new metastases have worse outcomes compared to those patients whose disease progresses due to growth of pre-existing lesions [11, 30].

Regarding toxicity, most patients in our series demonstrated acceptable tolerability of the eribulin treatment. The most frequent grade 3/4 toxicities were asthenia, neurotoxicity and neutropenia and were manageable. Remarkably, the proportion of grade 3 neutropenia was lower in our study than in pivotal trials (9.8% vs 23.4%), as well as grade 4 toxicities with 10.2% in our study (all of them neutropenia) versus 27.7% in the pooled analysis [8].

This study has several strengths and limitations. Our study reflects the everyday clinical practice use of eribulin and analyzes multiple variables such as BMI, age > 70 years and activity in patients with CNS metastases that have not been previously addressed and studied together in patients outside of a clinical trial. The major limitations of this study are the limited number of patients included and its retrospective nature without having a dedicated pre-planned design of patient management and data collection, which can lead to potential biases. It is worth noting that, during the study period, several clinical trials evaluating new targeted drugs in combination with eribulin were ongoing and those patients were not included in this study.

## Conclusion

The efficacy of eribulin in terms of OS and PFS observed in our study is similar to that reported in pivotal clinical trials. However, clinical benefit and ORR were higher, and significantly longer survival was seen in patients with HR-positive tumors, low burden disease and older than 70 years of age. Remarkably, less toxicity was found compared to previous studies and relevant CNS efficacy was observed. Despite the potential biases of this study, its results might be useful in clinical practice due to the limited existing data of eribulin’s efficacy and safety focused on age, BMI and CSN.

## Data Availability

The datasets used and/or analyzed during the current study are available from the corresponding author on reasonable request.

## Abbreviations

ABC: Advanced breast cancer
AE: Adverse events
BBB: Blood–brain barrier
BMI: Body mass index
CNS: Central nervous system
CR: Complete response
CTCAE 3.0: Common Terminology Criteria for Adverse Events
ECOG: Eastern Cooperative Oncology Group
EMT: Epithelial-mesenchymal transition
M1: Metastases
OS: Overall survival
PFS: Progression-free survival
PR: Partial response
RECIST 1.1: Response evaluation criteria in solid tumors
SD: Stable disease
TNBC: Triple-negative breast cancer
